# Policosanol fabrication from insect wax and optimization by response surface methodology

**DOI:** 10.1371/journal.pone.0197343

**Published:** 2018-05-15

**Authors:** Jinju Ma, Liyi Ma, Hong Zhang, Zhongquan Zhang, Youqiong Wang, Kai Li, Xiaoming Chen

**Affiliations:** Research Institute of Resources Insects, Chinese Academy of Forestry, Kunming, Yunnan, China; College of Agricultural Sciences, UNITED STATES

## Abstract

**Background:**

Insect wax is a famous biological resource for the role in economic production in China. Insect wax is a good source of policosanol, which may is a candidate supplement in foodstuff and pharmaceuticals that has important physiological activities. Therefore, this work aims to investigate a high-yield and rapid method for policosanol fabrication from insect wax.

**Results:**

The conditions for policosanol fabrication were optimized as follows: an oil bath temperature of 112.7°C and reductant dosage of 0.97 g (used for the reduction of 10.00 g of insect wax). The yield of policosanol reached 83.20%, which was 4 times greater than that of existing methods, such as saponification. The total content of policosanol obtained under the optimal conditions reached 87%. In other words, a high yield of policosanol was obtained from insect wax (723.84 mg/g), that was 55 times higher than that generated from beeswax-brown via saponification. The concentrations of metal residues in policosanol were within the limits of the European Union regulations and EFSA stipulation. The LD50 values for oral doses of insect wax and policosanol were both > 5 g/kg.

**Conclusion:**

Policosanol was fabricated via solvent-free reduction from insect wax using LiAlH_4_ at a high yield. The fabrication conditions were optimized. Policosanol and insect wax showed high security, which made them potential candidates as supplements in foods, pharmaceuticals and cosmetics. The rapid and high-yield method has great potential for commercial manufacturing of policosanol.

## Introduction

Chinese white wax scale (CWWS) (*Ericerus pela*) is a famous insect species because of its role in economic production in China, and these insects are widely distributed in most parts of China, Japan and the Korean peninsula, from the subtropics to temperate regions [1, 2]. CWWS have been bred in China for over one thousand years [3]. Insect wax is secreted by male *Ericerus pela* Chavanness, which mainly live on Chinese privet (*Ligustrum lucidum*) and Chinese ash (*Fraxinus chinesis*) [1]. Insect wax is lustrous, free of pollutants, and chemically stable [4]. The components of insect wax have been well defined and include cerylcerotate as the major component; a few esters consisting of even-numbered C26–C30 monobasic saturated fatty acids and their corresponding monobasic saturated fatty alcohols, which account for approximately 93–95% of its constituents; and a small amount of dissociative policosanols (1.0–1.5%) [4–6]. Insect wax has been used in printing, candle production and traditional medicine for a long time. Insect wax has also been used as a cosmetic material, insulative coating and fruit preservative [4]. Recent interest in insect wax has increased among the scientific and industrial communities, especially in the pharmaceutical field, because of its policosanol moieties, a family of high-molecular weight and saturated aliphatic primary alcohols (C20-C36). Hexacosanol, octacosanol and triacontanol are the main policosanols of interest due to their significant physiological activities. Hexacosanol exhibits neurotrophic activity in cultured neurons and attenuates the degeneration of cholinergic neurons after injury, in addition, hexacosanol at low doses reduces cell loss in neurodegenerative disorders such as Alzheimer’s disease [7, 8]. It is well known that octacosanol is an antifatigue agent [9, 10]. Many studies have shown that octacosanol is very effective in lowering low-density lipoproteins (LDLs) and increasing high-density lipoproteins (HDLs), and octacosanol also increases athletic performance, decreases the incidence of coronary heart disease, reduces the risk of coagulation in hematoblasts and offers cytoprotective effects [9–11]. As a plant growth regulator, triacontanol regulates photosynthesis, enzymatic activity and yield [11]. Policosanol is sold as a lipid-lowering supplement in more than 40 countries [12]. Currently, there is industrial interest in plant and insect matrices rich in policosanol for use in the development of functional foods and nutraceuticals [13]. Policosanol was originally isolated from sugar cane (*Saccharum officinarum L*.) [14]. Currently, policosanol is fabricated from beeswax, rice bran wax, sorghum kernel and wheat germ [15]. The content of wax ester in insect wax is higher than that in rice bran wax, which is 85–90% [16]. Insect wax is more pure compared with rice bran wax, which contains impurities such as unsaturated aliphatic acids, glycerides, free acids, gum impurities, hydrocarbons, and so on, in addition to wax esters [16–18]. Moreover, insect wax consists of cerylcerotate as the major component, and the main policosanols fabricated from rice bran wax are docosanol and tetracosanol [18]. Therefore, insect wax represents a potential alternative to rice bran wax for policosanol fabrication. However, evidence supporting such an approach is scarce.

Policosanol is mainly fabricated from sugar cane by hydrolytic cleavage of the wax and subsequent purification [19]. A solvent-alkali system is often used to hydrolyze rice bran wax, which is more difficult to hydrolyze than glycerides, to shorten the reaction time and improve the rate of hydrolysis [20]. Currently, policosanol is mostly fabricated via saponification [21, 22]. Wang et. al compared various fabrication methods, including dry saponification, saponification in alcohol and saponification in water, for the production of policosanol from rice bran wax, and the highest yield of policosanol was 28.0% [21]. When policosanol was fabricated from rice bran by saponification under high-intensity ultrasound, the yield of policosanol from the first bran layer was approximately 2% of the extract weight, and the wax from winterization treatment gave a yield of approximately 8% [22]. However, these methods are not preferred industrially due to their low efficiency and contribution to environmental pollution. Therefore, an alternative method for insect wax hydrolysis is needed to improve efficiency.

The objective of this study was to fabricate policosanol using solvent-free reduction of insect wax under atmospheric pressure, with a higher yield than that of saponification and to investigate the factors affecting policosanol fabrication. Currently, no other waxes have been reported to be reduced using LiAliH_4_ to prepare policosanol.

## Materials and methods

### Materials

Refined insect wax was supplied by Emei Insect Wax Institute (Emei, Sichuan, China), and unrefined insect wax was purchased from Zhaotong City, Yunnan Province, China. Lithium aluminium hydride, sodium hydroxide, anhydrous ethanol, hydrochloric acid, and chloroform were all of analytical reagent-grade. The individual policosanol standards of tetracosanol, hexacosanol, octacosanol and triacontanol were purchased from J&K (J&K Chemical Ltd., Beijing, China).

Ten female Sprague Dawley (SD) rats (Animal permit number: SCXK (liao) 2015–0001) were used for the acute oral toxicity studies. The rats were kept in a temperature-controlled room (20–26°C) with a 12 h light/dark cycle and relative humidity of 40–70%. Rats were acclimated to the Specific Pathogen Free (SPF) laboratory conditions for 25 days prior to the experiments. Water was available to the rats *ad libitum* throughout the study duration, except during the actual measurements. The experimental protocol was approved by the Ethics Committee of the Research Institute of Insect Resources of the Chinese Academy of Forestry (Kunming, China), and the animal experiments were performed in accordance with the Guide for the Care and Use of Experimental Animals.

### Fabrication of policosanol from insect wax via the solvent-free, “one-pot” reductive process

Pre-defined amounts of the reductant (LiAlH_4_) were added to insect wax at a specific oil bath temperature with continuous stirring to generate solid intermediates. Next, the solid intermediates were mixed with specific amounts of deionized water and chloroform and refluxed for 1 h at approximately 90°C. Boiling deionized water was added to the mud cake with continuous stirring after the solvent was recycled by rotary evaporation, and 10% hydrochloric acid was added until the mixture was slightly acidic. Subsequently, crude policosanol was repeatedly washed with boiling deionized water until its pH was approximately 7.0 and then vacuum-dried at 60°C.

### Study of the effect factors on policosanol fabrication by single-factor experiments

Unrefined insect wax was used in the selection of the reaction conditions to minimize the cost.

#### Oil bath temperature during reduction

Reduction of insect wax was conducted in oil baths at 90°C, 95°C, 100°C, 105°C and 110°C. The speed of reduction and total contents of the target products tetracosanol, hexacosanol, octacosanol and triacontanol were determined at each of the different temperatures.

#### Reductant dosage

To reduce 10.00 g of insect wax, 0.60 g, 0.70 g, 0.80 g, 0.90 g, 1.00 g, and 1.10 g of LiAlH_4_ were used, with at oil bath temperature of 105°C.

#### Washing times

Crude policosanol was washed one, two, three, four and five times with 10 volumes of boiling deionized water, and each sample measurement was conducted twice.

#### Bath ratio

The bath ratio in this test refers to the volume of chloroform as a fraction of the solid intermediates refluxed. The solid intermediates were mixed with 2, 4, 6, 8, and 10 volumes of chloroform and were then washed four times with deionized water.

### Optimization by response surface methodology (RSM)

Refined insect wax was used for the following optimization. Based on single-factor experiments, the Box-Behnken statistical design with 3 factors and 3 levels was used to evaluate the key factors and interactions between the oil bath temperature, reductant dosage and bath ratio on policosanol fabrication via solvent-free reduction of insect wax using Design-Expert 8.0.6. A design composed of 15 runs was developed, for which the nonlinear computer-generated quadratic model can be expressed as follows:

$$Y=a_{0}+a_{1}A+a_{2}B+a_{3}C+a_{12}AB+a_{13}AC+a_{23}BC+a_{11}A^{2}+a_{22}B^{2}+a_{33}C^{2}$$

where Y represents the response, a_0_ denotes the intercept, a_1_ to a_33_ are regression coefficients computed from the experimentally observed values of Y, and A, B and C are independent variables. The terms AB, AC and BC and A^2^, B^2^ and C^2^ represent the interaction and quadratic terms, respectively [23, 24]. S1 Table in the Supporting Information summarizes the coded values of the different variables. The oil bath temperature (A), reductant dosage (B) and bath ratio (C) were used for 15 experimental formulations. The total content of the target products tetracosanol, hexacosanol, octacosanol and triacontanol are listed in S2 Table of the Supporting Information.

### Characterization of the fabricated policosanol

#### GC-FID analyses

Gas chromatography—flame ionization detector (GC-FID) was used to determine the policosanol composition. The level of each aliphatic alcohol was analyzed using internal standards. GC separations were performed using a BD-5 capillary column (30 m × 0.25 mm i.d., film thickness of 0.25 μm). The constant temperature of the column was set at 290°C using highly purified nitrogen as the carrier gas at a column flow of 1.7 mL/min. The injector and detector temperatures were set at 300°C. The injection volume was 2 μL, and the split ratio was 1:10.

#### Metal residue detection

The metal residues including As, Pb, Hg, Cd and Al in the fabricated policosanol were determined according to the commission regulations of the European Union [25–27]. 0.4980 g of policosanol sample was digested by 5 mL of nitric acid and 2 mL of hydrogen dioxide solution (30%) at 140°C in the polytetrafluoroethylene inner pot for following detections. Then the concentrations of lead and cadmium were detected by graphite furnace atomic absorption spectrometry, and the detection conditions were as follows: 0.2 ~ 1.0 nm of slit, 5 ~ 7 mA of lamp current, 105 ~ 120°C of drying temperature for 20 s, 450°C of ashing temperature for 20 s, 283.3 nm (lead) or 228.8 nm (cadmium) of wavelength. The concentration of arsenic was detected by inductively coupled plasma mass spectrometry using argon as the carrier gas at a flow of 1.14 L / min. The concentration of mercury was detected by atomic fluorescence spectrometry, and the electric current of Hg hollow cathode lamp was 30 mA; the temperature of atomizer was 300°C. And the concentration of aluminum was detected by spectrophotometry at 640 nm of wavelength after the reaction with chrome azurol S and cetyltrimethylammonium bromide in the acetic acid—sodium acetate.

#### Acute oral toxicity studies of insect wax and policosanol

The experiments were conducted according to the Organization for Economic Co-operation and Development (OECD) Guideline 420 [28]. Insect wax and policosanol were added to corn oil and stirred evenly. Insect wax and policosanol were administered orally (5 g/kg) in a single dose to female rats (9 weeks, 238–248 g, n = 5 each group). Rats were fasted overnight prior to dosing and 3 h after treatment. The general behavior and clinical signs of toxicity of the rats (such as changes in skin, hair, eyes, mucous membranes, central nervous systems, etc.) were monitored continuously during the first 24 h (0.5, 1, 2, 4 and 12 h) and daily until day 14 after dosing. The body weights of the rats were measured on days 1, 7, and 14 [28]. The pathological anatomy of rats was investigated on the 15^th^ day after anesthesia by 2% pentobarbital sodium (50 mg/kg BW). The rest of the rats were euthanized by carbon dioxide suffocation on the day 15 after dosing.

## Results and discussion

### Study of the effect factors on policosanol fabrication by single-factor experiments

#### Effect of the oil bath temperature

**Fig 1:**
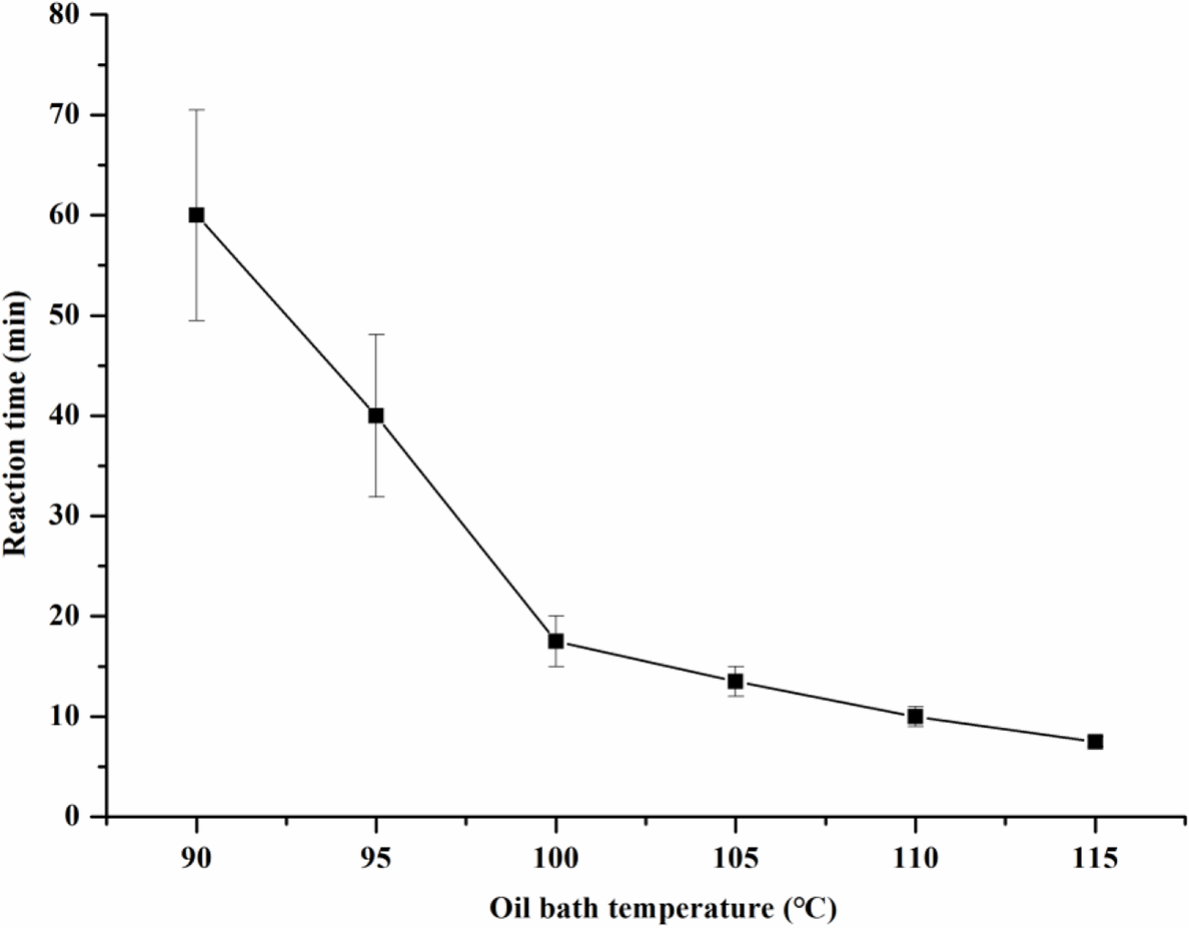
Effect of the oil bath temperature on reaction time.

The reaction speed and total contents of four target products obtained at different oil bath temperatures were compared. As seen in Table 1 and Fig 1, the temperature had a significant effect on the reaction speed. The reaction time was reduced from approximately 60.0 min to nearly 7.5 min when the oil bath temperature was increased to 115°C from 90°C. However, the reduction speed in oil baths at 120°C and above was rapid, resulting in poor control. The reductant LiAlH_4_ was degraded at approximately 130°C. The final total content of the four target products was maximal at 110°C. Therefore, oil bath temperatures of 105°C and 115°C were selected to optimize the reaction and minimize the cost.

**Table 1:** Effect of the oil bath temperature on policosanol fabrication.

Oil bath temperature/ °C	Tetracosanol (C24) %	Hexacosanol (C26)/%	Octacosanol (C28)/%	Triacontanol (C30)/%	Total ^a^ / %	Reaction time /min
90	6.39	29.56	15.70	1.93	53.58 ± 0.21	60.0 ± 10.5
95	5.75	31.01	15.30	2.18	54.24 ± 0.10	40.0 ± 8.1
100	5.88	30.23	17.34	2.65	56.10 ± 0.15	17.5 ± 2.5
105	5.88	30.85	18.06	2.49	57.28 ± 0.12	13.5 ± 1.5
110	6.20	31.75	19.00	2.68	59.63 ± 0.13	10.0 ± 1.0
115	5.57	30.96	18.86	2.34	57.73 ± 0.11	7.5 ± 0.5

^a^ Total content of the four policosanols (C24, C26, C28, C30).

#### Effect of reductant dosage

The influence of the reductant dosage on policosanol fabrication is illustrated in S1 Fig. Higher total contents of tetracosanol, hexacosanol, octacosanol and triacontanol were obtained using more than 0.70 g of LiAlH_4_.

**Fig 2:**
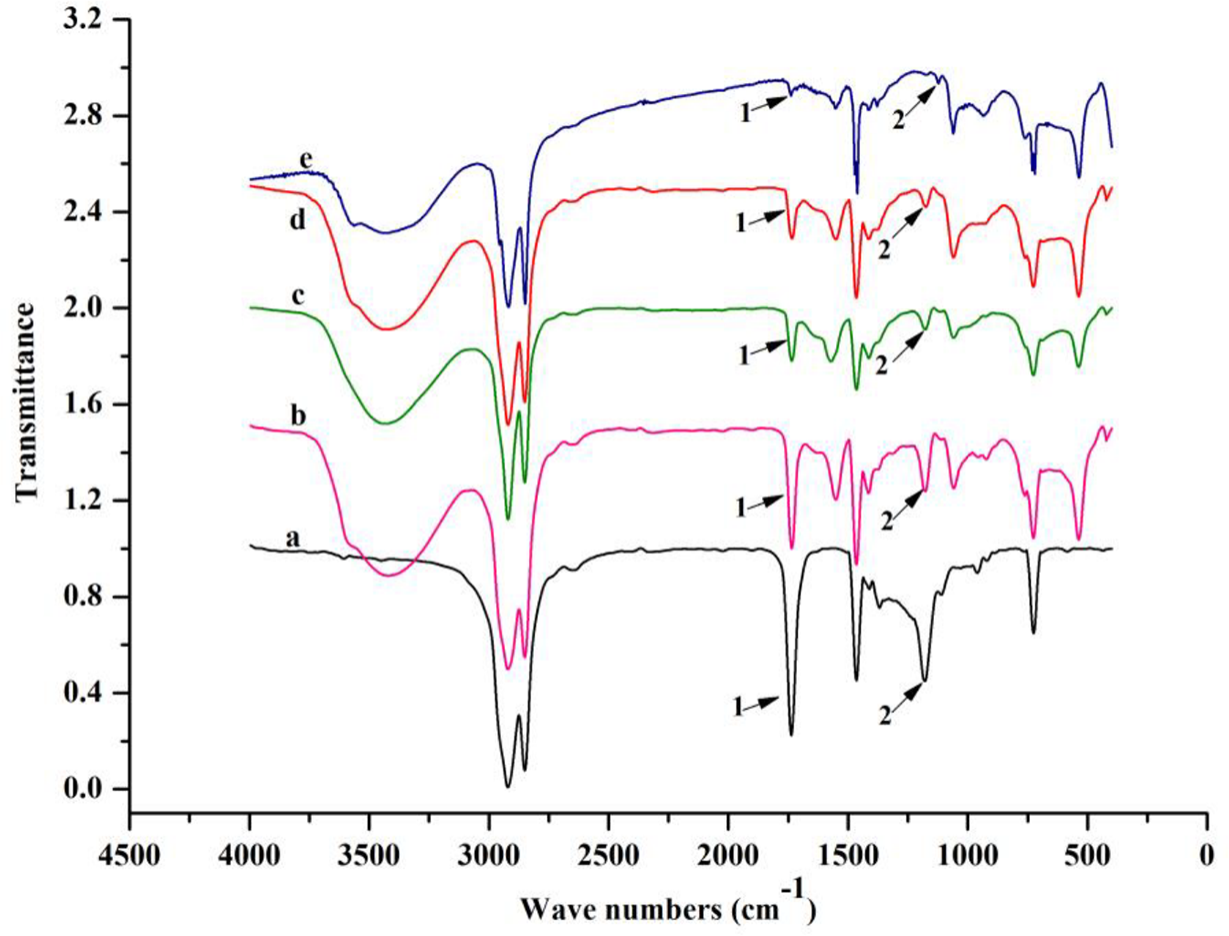
FTIR spectra of policosanol and insect wax. Insect wax (spectrum a), policosanol fabricated with 0.6 g of LiAlH_4_ (spectrum b), policosanol fabricated with 0.7 g of LiAlH_4_ (spectrum c), policosanol fabricated with 0.8 g of LiAlH_4_ (spectrum d), and policosanol fabricated with 0.9 g of LiAlH_4_ (spectrum e); peak 1, approximately 1733 cm^-1^, peak 2, approximately 1178 cm^-1^.

In addition, we obtained the FTIR spectra of insect wax and policosanol extracted using different quantities of reductant for qualitative analysis. As shown in Fig 2, compared with insect wax (spectrum a), the characteristic intensity peaks, including those of C = O at 1733 cm^-1^ (peak 1) and C(O)-O at 1178 cm^-1^ (peak 2), for policosanol fabricated with different dosages of reductant (spectra b, c, d, and e) were gradually weakened. This finding indicated that insect wax that was not reduced was diminished with increased levels of the reductant LiAlH_4_. The results also indicated that the reduction was not complete when the dosage of reductant was less than 0.80 g. Further, the new peak observed at 3427 cm^-1^ (spectra b, c, d, and e) was attributed to the stretching vibration of an alcoholic hydroxyl group, suggesting that insect wax was reduced to policosanol. Therefore, a range of 0.70 g-1.10 g of reductant was selected for optimization.

#### Effect of washing times

The crude policosanol mixture was washed one, two, three, four and five times with boiling deionized water to remove impurities (see S2 Fig). Washing repetition had almost no effect on the policosanol content but affected the levels of metal residues. Therefore, washing four times was considered appropriate based on the experimental levels of metal residues and cost minimization.

#### Effect of bath ratio

The bath ratio refers to the volume of chloroform as a fraction of the solid intermediates refluxed. The effect of the bath ratio on policosanol fabrication is presented in S3 Fig. Higher total contents of the four target products were obtained when the bath ratio was more than two, which may be due to incomplete dissolution of policosanol in 2 or fewer volumes of chloroform. The differences in the total contents obtained with bath ratios of 4, 6, 8, and 10 are shown in S3 Fig. Increasing the bath ratio raised the cost and difficulty of subsequent processing, and extremely low bath ratios resulted in poor output. Therefore, bath ratios of 4, 6, and 8 were selected for optimization.

### Optimization by response surface methodology

#### Analysis of variance (ANOVA)

Response surface methodology (RSM) is a powerful mathematic approach for building models, optimizing multifactor experiments, and analyzing the effects of multiple factors, alone or in combination, and is widely used in many fields since it produces large amounts information by performing a small number of experiments [29, 30]. ANOVA data are presented in S3 Table of the Supporting Information and suggest that the quadratic model was effective. The model “Prob > F” of less than 0.0500 implied that the model terms were significant. The R^2^ value of 0.9724 and R_adj_^2^ value of 0.9226 were in the accepted range [31], implying that 7.74% of the variation in the Y response could not be explained by this model. The model effectively predicted 92.26% of the total experimental data. The “Lack of Fit P-value” of 0.8408 implied that the lack of fit was insignificant, which indicated that the model used was fitted. Hence, this model was used to navigate the response surface design space. The comparison of the actual and predicted values is presented in S4 Fig. The 15 dots shown in S4 Fig correspond to the 15 groups of data presented in S2 Table. The shorter the distance from the point to the diagonal line, the more reliable the value. Therefore, most of the values were reliable as shown in S4 Fig. Normally, if the internally studentized standardized residual of one experimental point was located beyond the range of -2 to +2, it was considered to be abnormal, with a 95% confidence level. As seen in S5 Fig, there were no points lying outside the range of -2 to +2, indicating that all of the 15 points were credible and reliable.

**Fig 3:**
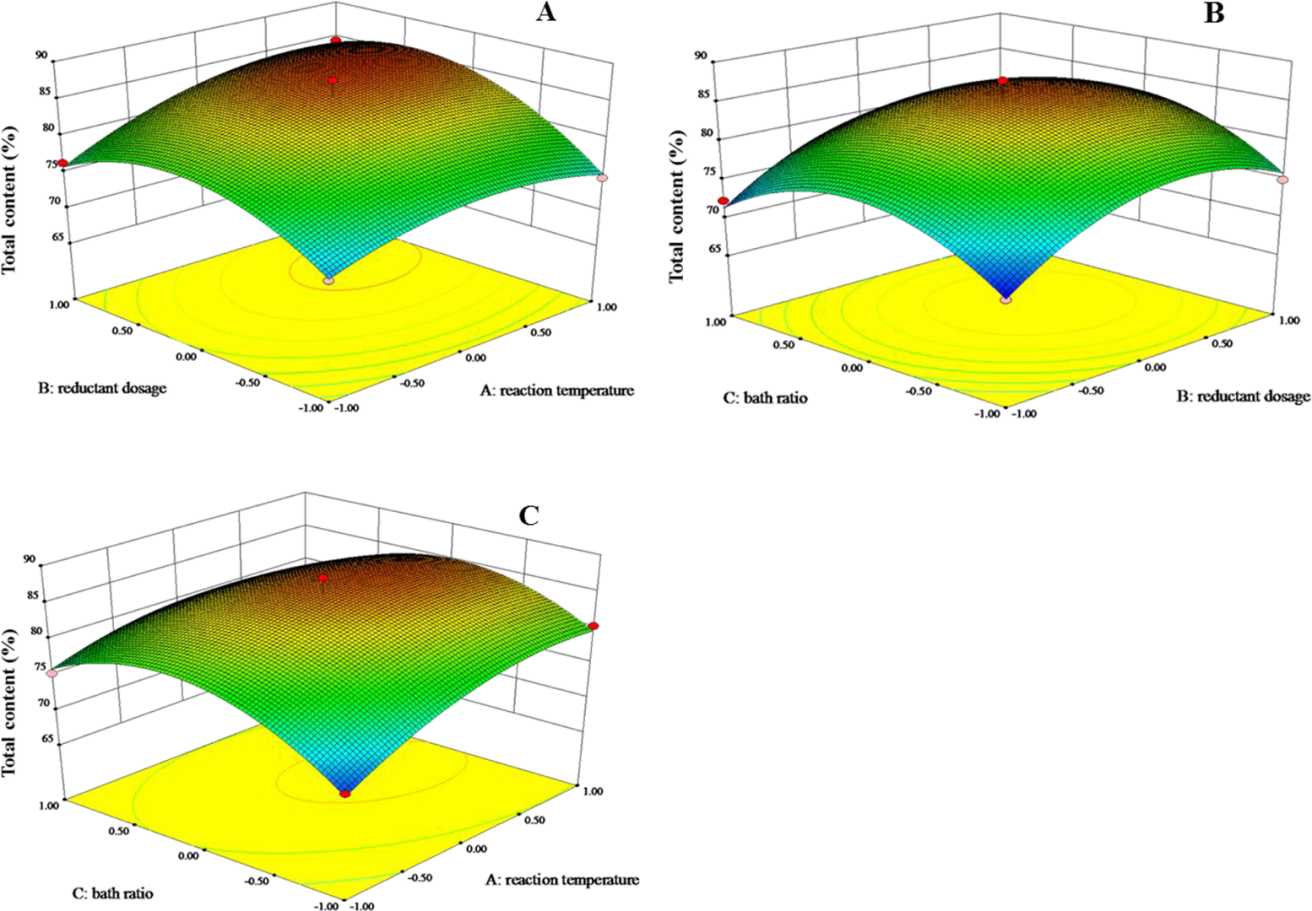
Response surface graph of the total content. (A) The effect of variables A-B (reaction time—reductant dosage); (B) effect of variables B-C (reductant dosage—bath ratio); (C) effect of variables A-C (reaction time—bath ratio).

The 3D-response surfaces indicating the superimposed effects of reaction temperature, reductant dosage and bath ratio are shown in Fig 3. Combined with the “Prob > F” value, the F value of the independent variables A, B, and C (in S3 Table, supporting information) and the 3D-response graphs (Fig 3), the influence of the three variables on the total content of policosanol was in the following order: reductant dosage > oil bath temperature > bath ratio. With an increasing oil bath temperature, the total content of the four target products first increased partially due to comprehensive reaction. However, when the oil bath temperature exceeded 112.7°C, LiAlH_4_ started to decompose due to an exothermic reaction, which reduced the total content.

#### Optimization of policosanol fabrication

**Fig 4:**
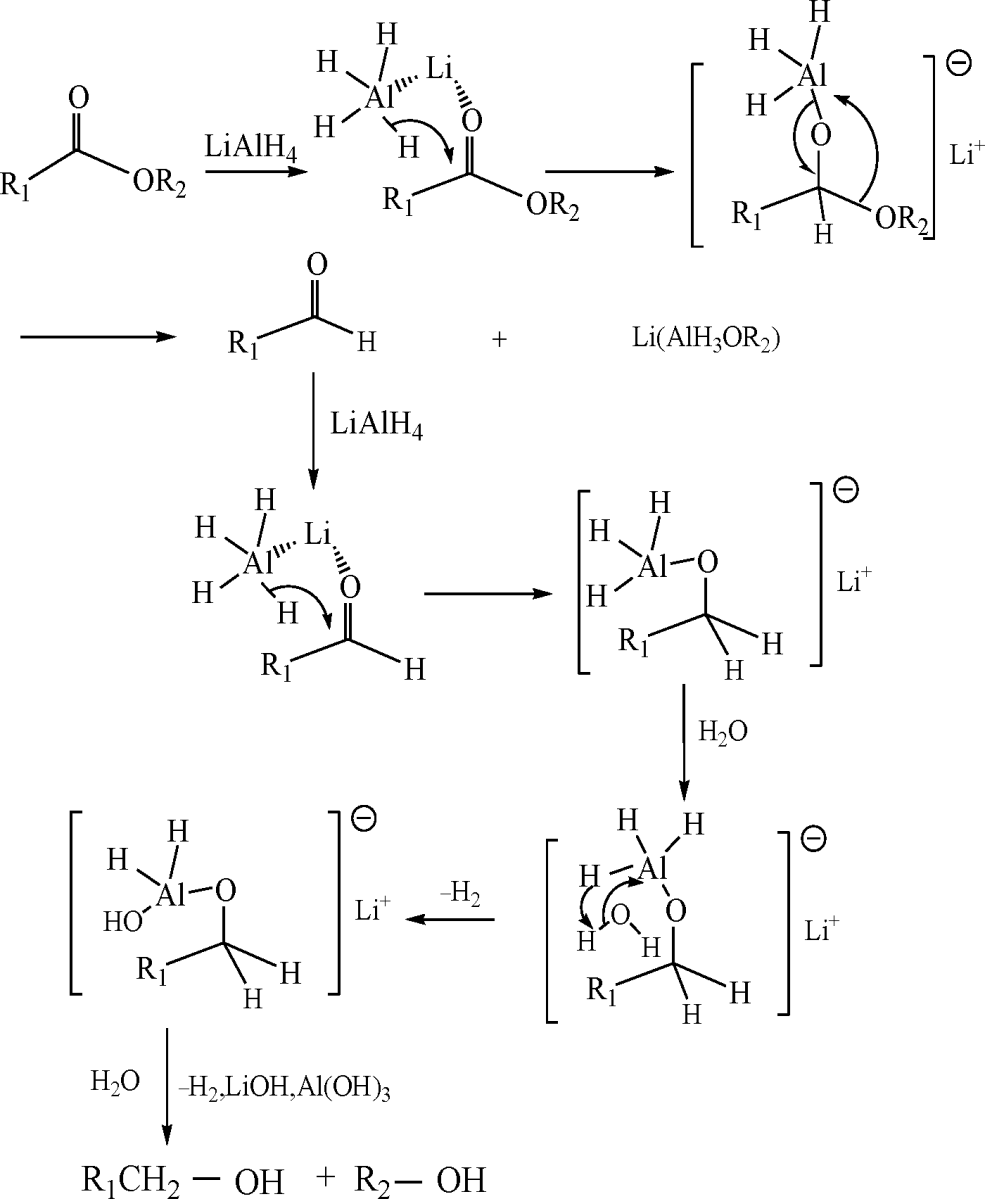
Reaction mechanism of the reduction of insect wax using LiAlH_4_ to produce policosanol.

Based on the Box-Behnken design, the conditions for policosanol fabrication were optimized as follows: an oil bath temperature of 112.7°C, reductant dosage of 0.97 g (used for reduction of 10.00 g of insect wax), and bath ratio of 6. To verify the predicted value, policosanol was fabricated under these conditions. The total content of the target products was 86.83%, which was very close to the predicted value (86.03%), demonstrating that optimization of the process parameters via response surface methodology was reasonable and stable, with practical significance. The total content of policosanol generated under optimal conditions reached 80% to 87%. The contents of tetracosanol, hexacosanol, octacosanol and triacontanol were 6% to 7%, 42% to 46%, 27% to 28%, and 5% to 6%, respectively. Additionally, the yield of policosanol was 83.20% under the optimal conditions. In other words, a high amount of policosanol was obtained via solvent-free reduction from insect wax (approximately 665.60 mg/g to 723.84 mg/g). Table 2 compares the policosanol yields and contents in this work with those of other fabrication methods. It is observed that the total content of policosanol obtained in this work was approximately 55 times higher than that of fabrication from beeswax-brown, in which beeswax is hydrolyzed by refluxing with 1.0 N NaOH in methanol for 30 min [15]. The yields of policosanol fabricated from rice bran wax via saponification in alcohol and water were 20.20% and 28.00%, respectively [21]. Obviously, the yield in this work was approximately 4 times that of the other fabrication methods. In terms of the reaction mechanism, one molecule of policosanol is gained via saponification from one molecule of wax ester, while, one molecule of wax ester is reduced by LiAlH_4_ into two molecules of policosanol (as seen in Fig 4), which indicates that the yield of the LiAlH_4_ process must be higher than that of saponification.

**Table 2:** Comparison of the policosanol yields and contents from this work and other fabrication methods from different samples.

Samples	Policosanol yield ^a^/ %	Tetracosanol /(mg/g)	Hexacosanol /(mg/g)	Octacosanol /(mg/g)	Triacontanol /(mg/g)	Total /(mg/g)
Beeswax-brown ^b^	—	2.60	1.70	2.00	5.70	12.00
Rice bran wax	20.20 ^c^	—	—	19.63 ^d^	30.62 ^d^	—
Rice bran wax	28.00 ^c^	—	—	10.78 ^d^	16.21 ^d^	—
Wheat straw ^b^	—	1.07 × 10^−2^	4.8 × 10^−3^	0.14	3.1 × 10^−3^	0.16
Sugar cane peel ^b^	-	7.70 × 10^−3^	2.30 × 10^−2^	0.22	1.60 × 10^−2^	0.27
Insect wax ^e^	43.20	—	—	—	—	—
Insect wax, this study	83.20	49.92–58.24	349.44–382.72	224.64–232.96	41.60–49.92	665.60–723.84

^a^ The total policosanol yields are directly expressed as Yield (%) = (m/M) × 100, where m is the weight of the final policosanol product (g), and M is the weight of the samples (g).

^b^ Data from Dunford et al. [15].

^c^ Data from Wang et al. [21].

^d^ Data calculated according to Wang et al [21].

^e^ Data were from Zhu, in which insect wax was saponified to prepare policosanol [32].

Further, the differences in the total content between the single-factor experiments and response surface methodology were attributed to the use of insect wax and refined insect wax in the two experiments, respectively, to minimize costs.

### Characterization of the fabricated policosanol

#### Gas chromatography

**Fig 5:**
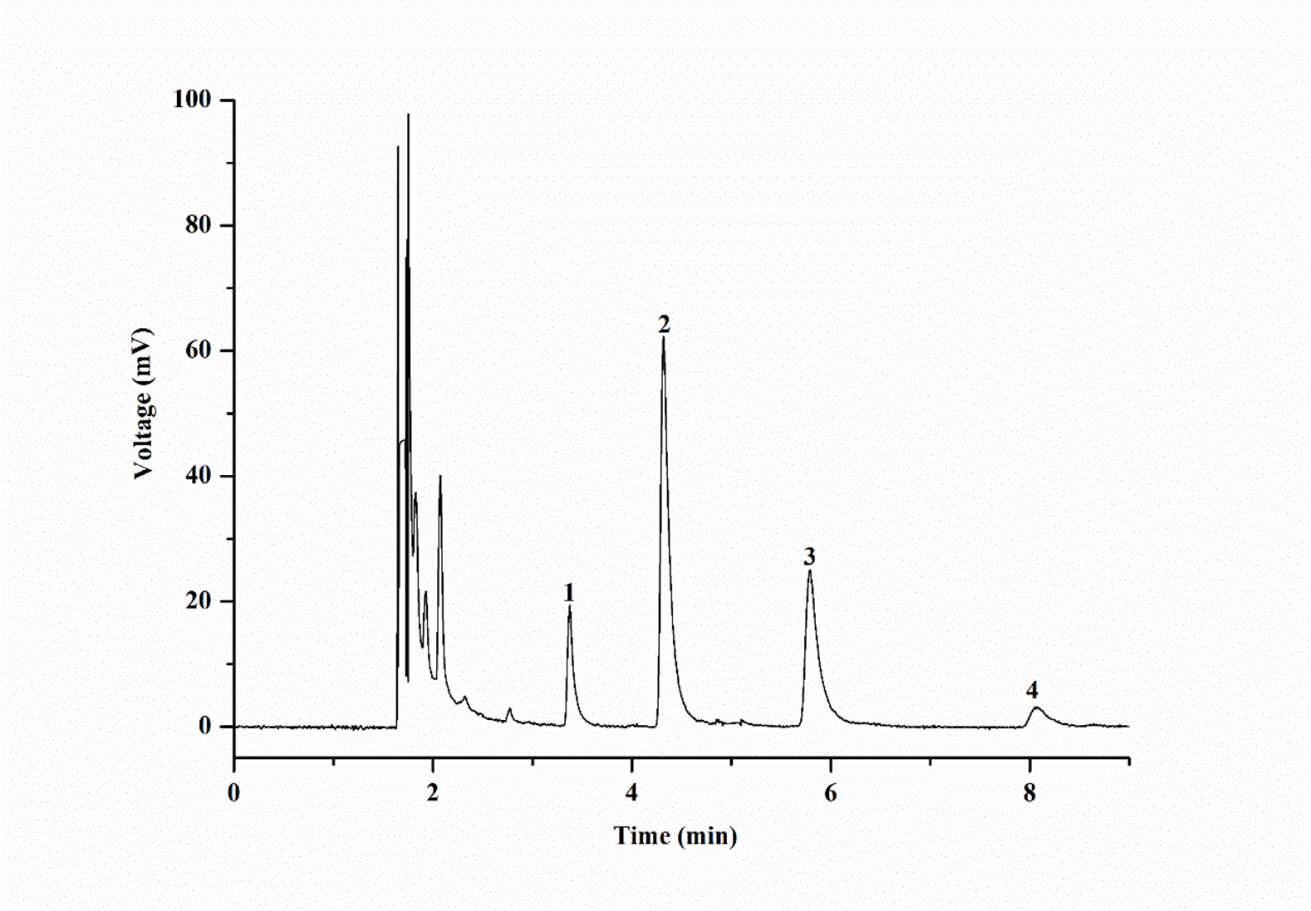
GC chromatogram of the policosanol mixture. Peaks: 1, tetracosanol; 2, hexacosanol; 3, octacosanol; 4, triacontanol.

The policosanol mixture obtained included a series of C20-C30 saturated alcohols. The contents of the target products tetracosanol, hexacosanol, octacosanol and triacontanol were determined using internal standards. All of the products had a retention time exceeding 3 min due to the smooth baseline and fast processing time (Fig 5).

#### Metal residues detection

The concentrations of metal residues in policosanol were identified and are displayed in Table 3. The results indicated that the concentrations of As, Pb, Hg and Cd in policosanol were all within the limits of the European Union regulations for foodstuffs. On the surface, the concentration of Al (31.700 mg/kg) exceeded the limit. However, the European Food Safety Authority (EFSA) established a tolerable weekly intake (TWI) of 1 mg of aluminum/kg bw/week. The panel also noted that the estimated daily dietary exposure to aluminum in the general population, as assessed in several European countries, varied from 0.2 to 1.5 mg/kg bw/week at the mean and was up to 2.3 mg/kg bw/week in highly exposed consumers [33]. Meanwhile, Borg reported that policosanol (1 mg/kg per day) protected central neurons from neurotoxic damage [34]. It was reported that policosanol (2 mg/kg per day) showed important physiological activity [35]. Oral administration of policosanol (35 mg/kg per day, for 14 d) can significantly ameliorate 6-OHDA-induced motor impairment in rats [36]. Markrack reported that the LDL cholesterol of thirty thousand people could be reduced by 20–25% after intake of policosanol for 6 months (10 mg per day) [37]. That is very small amounts of policosanol can exert important physiological activities. Thus, the concentration of aluminum in policosanol at a dosage that meets the demands of medicine, health care, and so on is within a TWI of 1 mg/kg bw/week. The results show that policosanol fabricated via reduction from insect wax using LiAlH_4_ can be safely applied to health products, medicine, and so on, which suggests the practical value of the optimized conditions reported in this study. The rapid and high-yield method has great potential for commercial manufacture of policosanol.

**Table 3:** The metal concentrations in fabricated policosanol.

Items	Detected concentrations (mg/kg)	Maximum levels of contaminants in foods (mg/kg)
As	0.054	0.100 ^b^
Pb	<0.100	3.000 ^a^
Hg	<0.010	0.100 ^a^
Cd	<0.050	1.000 ^a^
Al	31.700	10.000 ^c^

^a^ Data are from the literature (food supplements) [38].

^b^ Data are from the literature [39].

^c^ Data are from the literature [40, 41].

#### Acute oral toxicity of insect wax and policosanol

It has been reported that the LD50 can be used to evaluate relatively acute hazards of drugs [42]. Oral administration of insect wax and policosanol both produced no lethal effects or toxicity over the 14 days of the experiment. There were no obvious changes in weight gain or clinical signs or gross pathological changes in any of the rats during the acute oral toxicity test. The LD50 values for the oral dose of insect wax and policosanol were both > 5 g/kg. According to the OECD criteria, the product is classified in the GHS 5 category, of which the LD50 is between 2 and 5 g/kg [43, 44]. Interestingly, it has been documented that if no clinical signs of toxicity are observed and no death registered up to a dose of 5 g/kg during acute toxicity studies, then the substance can be considered to be non-toxic [45]. Hence, insect wax and policosanol fabricated from insect wax are non-toxic, which make them potential candidates for supplements in foods, pharmaceuticals and cosmetics.

## Conclusions

Policosanol was fabricated via solvent-free and “one-pot” reduction from insect wax. The Box-Behnken response surface analyses illustrated that the influences of the three variables on policosanol fabrication were in the following order: reductant dosage > oil bath temperature > bath ratio. The optimized conditions for policosanol fabrication were as follows: reduction of 10.00 g of insect wax using 0.97 g of LiAlH_4_ at an oil bath temperature of 112.7°C under normal pressure, followed by refluxing in 6 volumes of chloroform and deionized water and washing the crude policosanol four times with 10 volumes of deionized water. The yield of policosanol reached 83.20%, which was approximately 4 times that of existing fabrication methods, such as saponification. The total content of policosanol under the optimal conditions reached 80% to 87%. That is a high content of policosanol was obtained from insect wax (approximately 665.60 mg/g to 723.84 mg/g). The levels of tetracosanol, hexacosanol, octacosanol and triacontanol were 6% to 7%, 42% to 46%, 27% to 28%, 5% to 6%, respectively. In addition, the concentrations of metal residues in policosanol were within the limits of the European Union regulations and EFSA stipulation. The LD50 values for the oral dose of insect wax and policosanol were both > 5 g/kg, which indicates that insect wax and policosanol are non-toxic.

## Supporting information

S1 Fig Effect of the reductant dosage on policosanol fabrication. (TIF)

S2 Fig Effect of the washing times on policosanol fabrication. (TIF)

S3 Fig Effect of the bath ratio on policosanol fabrication. (TIF)

S4 Fig The comparison between actual and predicted values. (TIF)

S5 Fig The internally studentized standardized residuals of each experimental point. (TIFF)

S1 Table The coded values of variables in Box-Behnken design for optimization. (DOCX)

S2 Table The observed responses in Box-Behnken design for optimization. (DOCX)

S3 Table The response surface quadratic model: Regression analysis and ANOVA. (DOCX)
