# Supplementary figures and images for: Policosanol fabrication from insect wax and optimization by response surface methodology
Source: PLoS One. 2018 May 15;13(5):e0197343. doi: 10.1371/journal.pone.0197343 (PMC5953464; doi:10.1371/journal.pone.0197343)

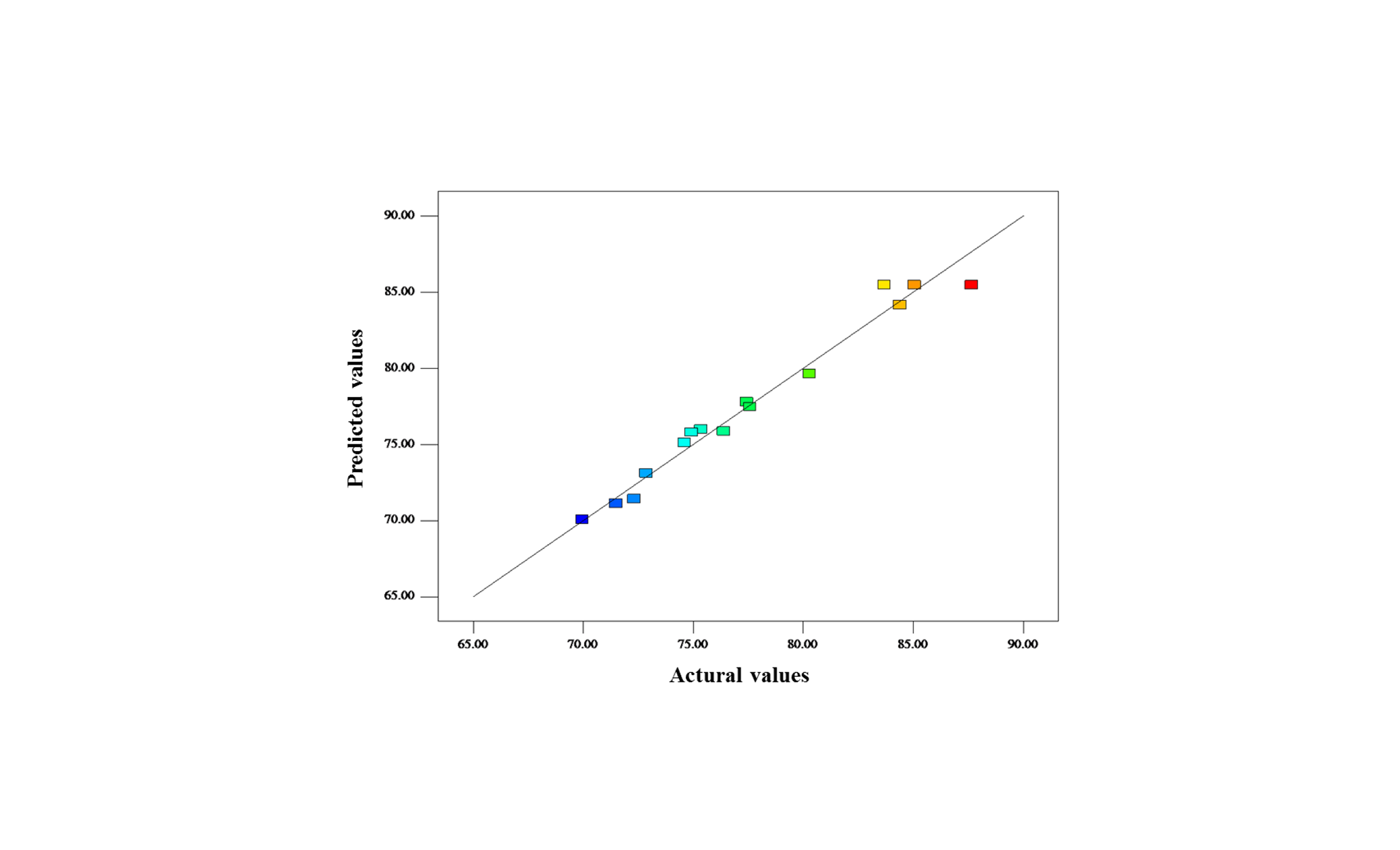

Supplement: S4 Fig — (TIF) [file pone.0197343.s004.tif]

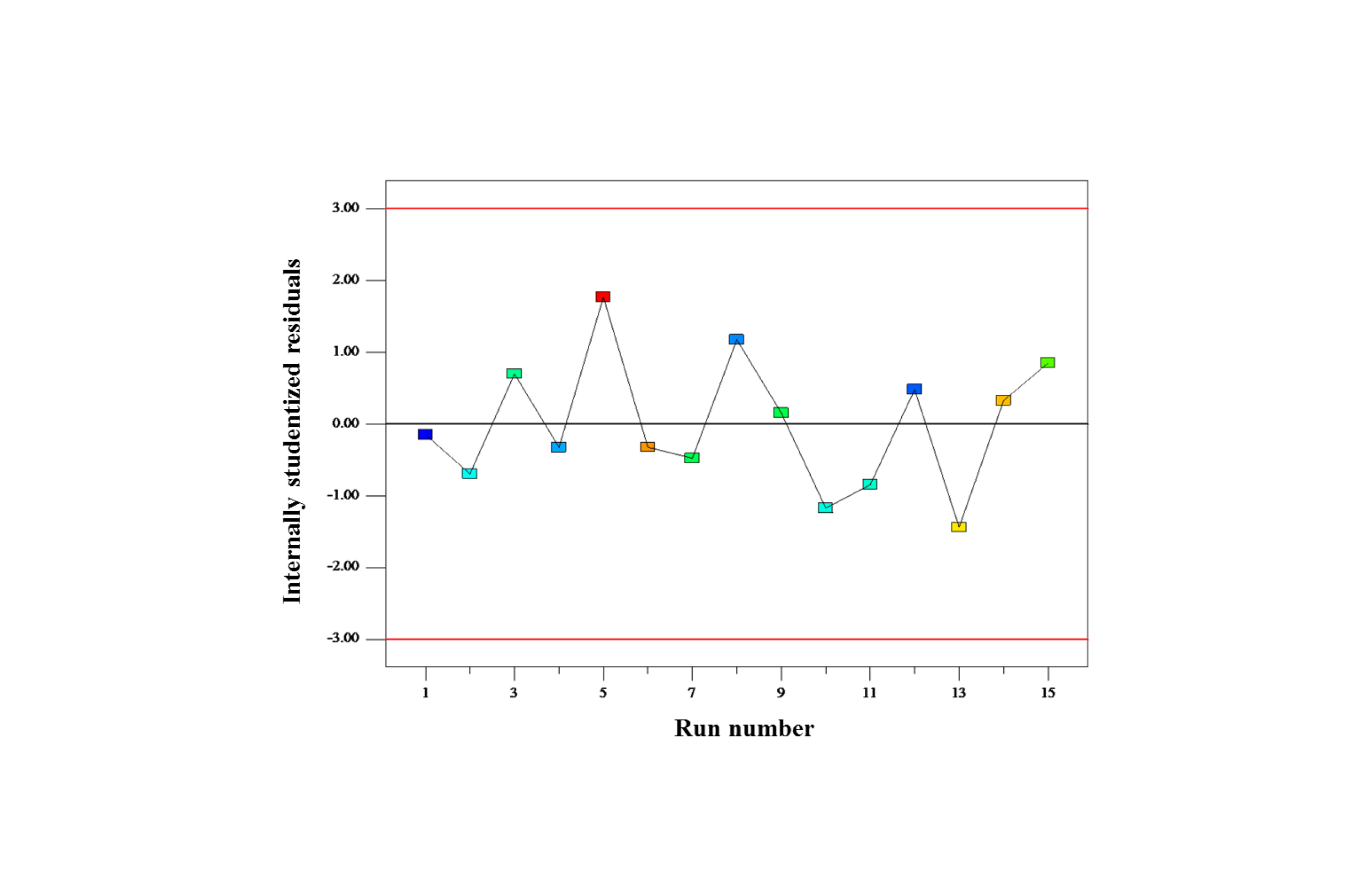

Supplement: S5 Fig — (TIFF) [file pone.0197343.s005.tiff]
