# Supplementary material for: Policosanol fabrication from insect wax and optimization by response surface methodology
Source: PLoS One. 2018 May 15;13(5):e0197343. doi: 10.1371/journal.pone.0197343 (PMC5953464; doi:10.1371/journal.pone.0197343)
Supplement: S1 Table — (DOCX) [file pone.0197343.s006.docx]

**Supplemental Tables**

S1 Table The coded values of variables in Box-Behnken design for optimization

| Independent variables |  | Levels |  |
| --- | --- | --- | --- |
|  | -1 | 0 | 1 |
| (A) oil bath temperature /ºC | 105 | 110 | 115 |
| (B) dosage /g | 0.70 | 0.90 | 1.10 |
| (C) bath ratio/times | 4 | 6 | 8 |
