# Supplementary material for: Policosanol fabrication from insect wax and optimization by response surface methodology
Source: PLoS One. 2018 May 15;13(5):e0197343. doi: 10.1371/journal.pone.0197343 (PMC5953464; doi:10.1371/journal.pone.0197343)
Supplement: S2 Table — (DOCX) [file pone.0197343.s007.docx]

**Supplemental Tables**

S2 Table The observed responses in Box-Behnken design for optimization

| Batch | Oil bath temperature | Dosage | Bath ratio | Total content / % |
| --- | --- | --- | --- | --- |
| 5 | -1.00 | 0.00 | -1.00 | 71.49 |
| 9 | 0.00 | -1.00 | -1.00 | 69.95 |
| 8 | 1.00 | 0.00 | 1.00 | 77.43 |
| 15 | 0.00 | 0.00 | 0.00 | 87.65 |
| 1 | -1.00 | -1.00 | 0.00 | 72.86 |
| 3 | -1.00 | 1.00 | 0.00 | 76.39 |
| 11 | 0.00 | -1.00 | 1.00 | 72.31 |
| 6 | 1.00 | 0.00 | -1.00 | 80.28 |
| 14 | 0.00 | 0.00 | 0.00 | 85.06 |
| 10 | 0.00 | 1.00 | -1.00 | 74.91 |
| 13 | 0.00 | 0.00 | 0.00 | 83.68 |
| 12 | 0.00 | 1.00 | 1.00 | 77.58 |
| 4 | 1.00 | 1.00 | 0.00 | 84.40 |
| 7 | -1.00 | 0.00 | 1.00 | 75.35 |
| 2 | 1.00 | -1.00 | 0.00 | 74.60 |
