# Supplementary material for: Policosanol fabrication from insect wax and optimization by response surface methodology
Source: PLoS One. 2018 May 15;13(5):e0197343. doi: 10.1371/journal.pone.0197343 (PMC5953464; doi:10.1371/journal.pone.0197343)
Supplement: S3 Table — (DOCX) [file pone.0197343.s008.docx]

**Supplemental Tables**

S3 Table The response surface quadratic model: regression analysis and ANOVA

| Source | Sum of Squares | df | Mean Square | F value | P-value  Prob >F |
| --- | --- | --- | --- | --- | --- |
| model | 404.64 | 9 | 44.96 | 19.54 | 0.0022 |
| A | 53.15 | 1 | 53.15 | 23.10 | 0.0049 |
| B | 69.38 | 1 | 69.38 | 30.16 | 0.0027 |
| C | 4.56 | 1 | 4.56 | 1.98 | 0.2182 |
| AB | 9.83 | 1 | 9.83 | 4.27 | 0.0936 |
| AC | 11.26 | 1 | 11.26 | 4.89 | 0.0779 |
| BC | 0.024 | 1 | 0.024 | 0.010 | 0.9226 |
| A^2^ | 32.69 | 1 | 32.69 | 14.21 | 0.0130 |
| B^2^ | 108.68 | 1 | 108.68 | 47.24 | 0.0010 |
| C^2^ | 148.90 | 1 | 148.90 | 64.73 | 0.0005 |
| Residual | 11.50 | 5 | 2.30 |  |  |
| Lack of Fit | 3.38 | 3 | 1.13 | 0.28 | 0.8408 |
| Pure Error | 8.12 | 2 | 4.06 |  |  |
| Corrected Total | 416.14 | 14 |  |  |  |
| R^2^=0.9724 | | R^2^_adj_=0.9226 | | CV=1.95% | |
